# Longitudinal Diffusion Tensor Imaging in Frontotemporal Dementia

**DOI:** 10.1002/ana.24296

**Published:** 2014-11-17

**Authors:** Colin J Mahoney, Ivor J A Simpson, Jennifer M Nicholas, Phillip D Fletcher, Laura E Downey, Hannah L Golden, Camilla N Clark, Nicole Schmitz, Jonathan D Rohrer, Jonathan M Schott, Hui Zhang, Sebastian Ourselin, Jason D Warren, Nick C Fox

**Affiliations:** 1 Dementia Research Centre, UCL Institute of Neurology, University College LondonLondon, United Kingdom; 2 Centre for Medical Image Computing, University College LondonLondon, United Kingdom; 3 London School of Hygiene and Tropical MedicineLondon, United Kingdom

## Abstract

**Objective:**

Novel biomarkers for monitoring progression in neurodegenerative conditions are needed. Measurement of microstructural changes in white matter (WM) using diffusion tensor imaging (DTI) may be a useful outcome measure. Here we report trajectories of WM change using serial DTI in a cohort with behavioral variant frontotemporal dementia (bvFTD).

**Methods:**

Twenty-three patients with bvFTD (12 having genetic mutations), and 18 age-matched control participants were assessed using DTI and neuropsychological batteries at baseline and ∼1.3 years later. Baseline and follow-up DTI scans were registered using a groupwise approach. Annualized rates of change for DTI metrics, neuropsychological measures, and whole brain volume were calculated. DTI metric performances were compared, and sample sizes for potential clinical trials were calculated.

**Results:**

In the bvFTD group as a whole, rates of change in fractional anisotropy (FA) and mean diffusivity (MD) within the right paracallosal cingulum were greatest (FA: −6.8%/yr, *p* < 0.001; MD: 2.9%/yr, *p* = 0.01). *MAPT* carriers had the greatest change within left uncinate fasciculus (FA: −7.9%/yr, *p* < 0.001; MD: 10.9%/yr, *p* < 0.001); sporadic bvFTD and *C9ORF72* carriers had the greatest change within right paracallosal cingulum (sporadic bvFTD, FA: −6.7%/yr, *p* < 0.001; MD: 3.8%/yr, *p* = 0.001; *C9ORF72*, FA: −6.8%/yr, *p* = 0.004). Sample size estimates using FA change were substantially lower than neuropsychological or whole brain measures of change.

**Interpretation:**

Serial DTI scans may be useful for measuring disease progression in bvFTD, with particular trajectories of WM damage emerging. Sample size calculations suggest that longitudinal DTI may be a useful biomarker in future clinical trials.

The behavioral variant of frontotemporal dementia (bvFTD) is a common cause of early onset dementia with progressive decline in personality and interpersonal skills, with gradual emergence of abnormal behaviors including apathy, obsessionality, and loss of empathy.^1^ Potential disease-modifying therapies for neurodegenerative disease are now emerging, creating an urgent need to develop biomarkers with improved accuracy to detect and monitor disease progression in bvFTD, not least because a high proportion of cases have a genetic basis, making presymptomatic intervention a real prospect.^2^

To date, longitudinal magnetic resonance imaging (MRI) has been shown to be a useful biomarker in neurodegenerative diseases, given its wide availability, ease of interpretation, and sensitivity in detecting change (most typically in brain volume) over time. Previous longitudinal imaging studies of bvFTD have used structural MRI to measure rates of whole brain and ventricular change.^3^–^6^ A limitation of some of these studies has been the tendency to measure rates of whole brain atrophy, rather than regionally based measures. This is significant, as whole brain techniques may be insensitive to the focal losses often seen in bvFTD. In addition, volumetric MRI may miss microstructural damage and may not provide sufficient sensitivity to detect meaningful change in individuals with slowly progressive forms of bvFTD, or in presymptomatic individuals with little macroscopic brain atrophy.^2^,^7^

A further problem in tracking progression in bvFTD is its broad pathological and clinical heterogeneity. Predicting underlying pathology on clinical or radiological grounds remains challenging. As such, the use of current clinical or neuroimaging measures to evaluate treatments, which will likely target specific molecular pathologies, is problematic. A number of recent reports have proposed a common "network-led" framework to understand how neurodegenerative diseases evolve.^8^,^9^ Selective molecular vulnerability of critical brain networks such as the Salience Network has been proposed in bvFTD.^10^ Following an initial insult to these brain networks, neurodegeneration may propagate across large-scale distributed brain networks. The emergence of sensitive neuroimaging techniques, such as diffusion tensor imaging (DTI), allows us to image the structural connections within these brain networks in vivo.

This study aims to address these issues by investigating longitudinal white matter change in key white matter structures in a group of patients with bvFTD. We also investigate the utility of DTI as a potential biomarker for clinical trials by comparing DTI measures of change with established MRI and neuropsychological measures and estimate sample size requirements for potential future clinical trials.

## Subjects and Methods

### Study Participants

Patients were recruited from 2009 to 2014 as part of a prospective study tracking disease progression in patients suspected to have frontotemporal lobar degeneration at the Specialist Cognitive Disorders Clinic, National Hospital for Neurology and Neurosurgery, London, United Kingdom. Patients who met current consensus criteria^1^ for a diagnosis of either probable or definite bvFTD and had 2 clinical and neuropsychological assessments and MRI scans (to include both T1-volumetric and DTI sequences) a minimum of 6 months apart were considered for study inclusion. All affected individuals provided a DNA sample for genetic analysis and were screened for mutations in microtubule-associated protein tau *(MAPT),* progranulin *(PGRN),* and chromosome 9 open-reading frame 72 *(C9ORF72)* genes. Twenty-three participants were identified as fulfilling inclusion criteria. Four participants were not included in the DTI analysis for the following reasons: 2 had significant artifact on follow-up DTI scans, 1 had an incomplete sequence due to scanner intolerance, and another had a sphenoid wing meningioma. Eighteen cognitively normal age- and gender-matched participants, with no history of psychopathy, stroke, myocardial infarct, or peripheral vascular disease, underwent the same test batteries as those with bvFTD. Each study participant underwent a battery of neuropsychological tests at baseline and follow-up, including assessments of general intellectual functioning, verbal and visual memory, naming, visuospatial and visual perception, calculation, and executive function. Social cognition was assessed using abbreviated versions of the emotion recognition (first 14 items) and social inference (first 9 items) subsets of the Awareness of Social Inference Test.^11^

### MRI Acquisition

Serial MRI scans were performed on the same Siemens Trio (Siemens, Erlangen, Germany) 3T MRI scanner using a 32-channel phased array head coil. Two 64-direction DTI sequences were acquired and averaged using a single-shot, spin-echo echo planar imaging sequence (55 contiguous axial 2.5mm slices with 240mm field of view and 96 × 96 matrix, yielding 2.5mm isotropic voxels; repetition time = 6,800 milliseconds, echo time = 91 milliseconds; b value = 1000 s/mm^2^). Field maps, b = 0 s/mm^2^ images, and a sagittal 3-dimensional magnetization prepared rapid gradient echo T1-weighted volumetric MRI (echo time/repetition time/inversion time = 2.9/2,200/900 milliseconds, dimensions = 256 × 256 × 208, voxel size = 1.1 × 1.1 × 1.1mm) were also acquired. Following visual inspection for artifacts, field map–based unwarping was applied to the diffusion-weighted images and they were affine-aligned to the average b0 image using FLIRT within the FMRIB Software Library (FSL v5.0.1 reference FSL) to correct for motion and eddy currents.^12^ Diffusion-weighted volumes were then combined and tensor fitting completed using CAMINO.^13^

### DTI Registration and Regions of Interest Ascertainment

**Figure 1:**
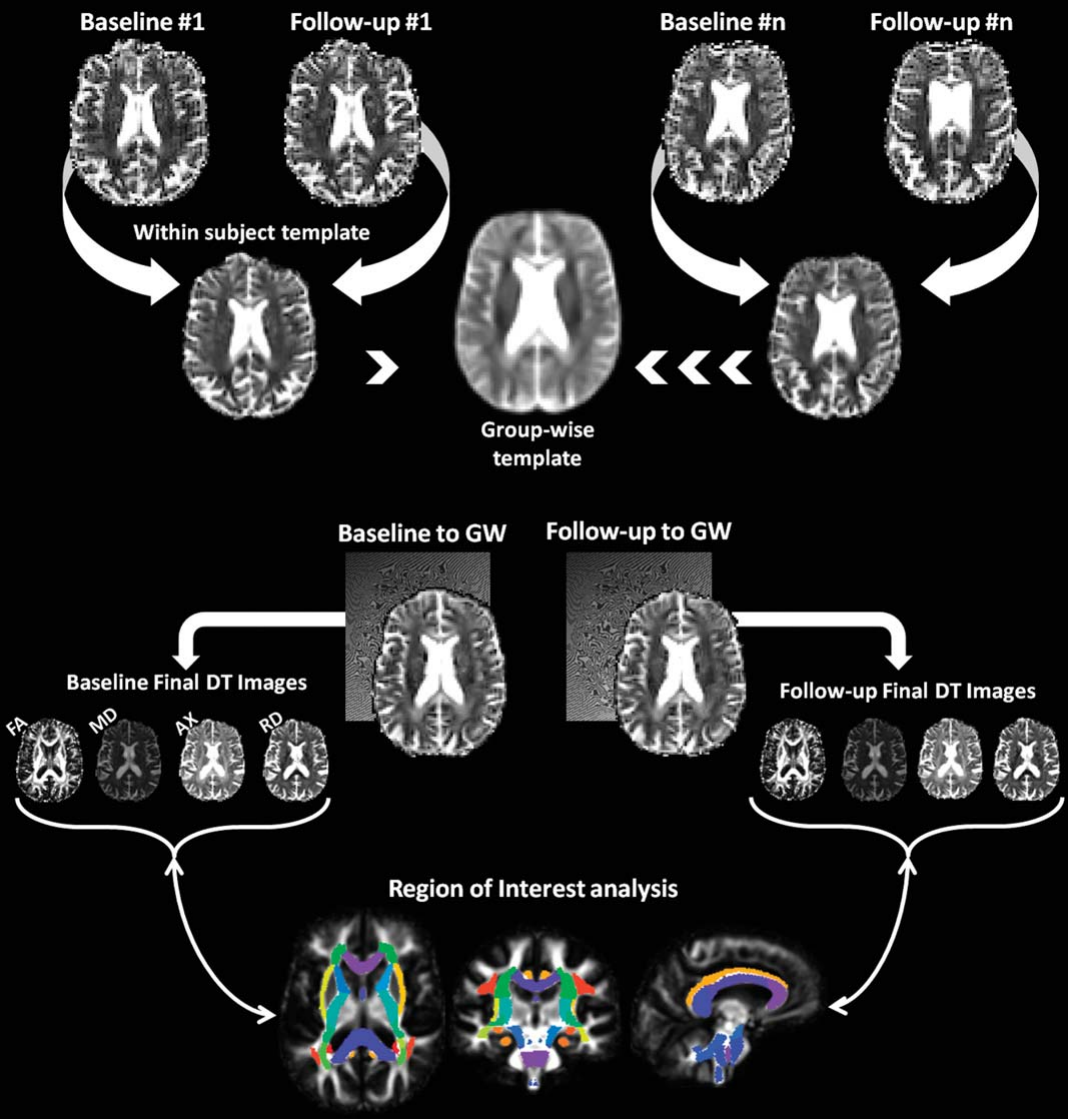
Overview of processing pipeline for longitudinal diffusion tensor imaging analysis. AX = axial diffusivity; DT = diffusion tensor; FA = fractional anisotropy; GW = group-wise; MD = mean diffusivity; RD = radial diffusivity. [Color figure can be viewed in the online issue, which is available at http://www.annalsofneurology.org.]

Registration of DTI images was carried out using a previously published method carried out on the same magnetic resonance scanner (see Fig 1 for an overview).^14^ This method has also been shown to have good reproducibility of DTI metrics when performing repeated DTI measurements on the same subject. Tensor-based registration was performed using the DTI-TK (http://dti-tk.sourceforge.net) software package,^15^ which uses the full diffusion tensor information to drive the registration and improve the alignment of white matter structures.^16^ Within-subject DTI templates are created using an iterative process of initial rigid registration, followed by nonlinear registration. This process is repeated using the intrasubject templates to create an intersubject groupwise template. A single deformation field was estimated for each original image to the groupwise template by combining the deformations fields from the intra- and intersubject registration stages. This facilitates a single interpolation of the original DTI images to the intersubject groupwise mean. Maps of fractional anisotropy (FA), mean diffusivity (MD), axial diffusivity (AX), and radial diffusivity (RD) were then created for each registered diffusion tensor image in the groupwise space.

To derive anatomically specific measures of diffusion change, the ICBM-DTI-81 white matter atlas was linearly and nonlinearly registered to the final FA template using FLIRT and FNIRT from FSL, and regions of interest, which included genu, body, and splenium of the corpus callosum, bilateral uncinate fasciculus, parahippocampal (ventral) cingulum bundle, paracallosal (dorsal) cingulum bundle, corticospinal tract, superior cerebellar peduncle, and fornix, were located using the white matter labels. Binary masks of each region were generated using a threshold of 50% on the white matter probability map with a further 1mm erosion around the boundary of each mask to provide high anatomical specificity. A visual inspection of each mask was performed to ensure appropriate coverage. The uncinate fasciculus did not undergo erosion, as this would have limited the mask size, reducing sensitivity to detect change in this tract.

### Volumetric Analysis

Volumetric image analysis was performed using a rapid, semiautomated segmentation technique yielding a brain region separated from surrounding cerebrospinal fluid, skull, and dura. Serial scans were aligned and volume change calculated directly using the boundary shift integral (BSI).^17^ BSI-derived whole brain volume changes, the brain boundary shift integral (BBSI), were expressed as annualized volume change as a percentage of the baseline brain volume.

### Statistical Analysis and Sample Size Estimates

Statistical analyses were carried out using Stata 12 (Statacorp, College Station, TX). Cross-sectional DTI metric data were compared between disease and cognitively normal groups using a linear regression model adjusting for age, gender, and disease duration. Mixed-effects linear regression models with random intercept were used to compare longitudinal change between groups for each DTI metric and region of interest, adjusting for age, gender, and disease duration. For longitudinal models log of each DTI metric was the dependent variable, with disease group, time from baseline scan in years, and interaction between disease group and time included to provide estimates of differences in the rate of change as a percentage per year. This methodology was also used to compare cross-sectional and longitudinal neuropsychological data between groups.

To determine the accuracy, sensitivity, and specificity of each DTI metric in classifying individual participants into separate groups (bvFTD or control), receiver operating characteristic curves were constructed using either raw DTI metric data (for baseline measurement) or the estimated mean difference in the rate of change for each diffusivity metric (for longitudinal measurement). Areas under the curve (AUCs) were calculated for regions of interest, which were significantly different when compared with controls.

Estimation of sample sizes for future trials were calculated (using Stata) with 80% power and 5% 2-tailed significance using the mean difference in the rate of change between groups for each DTI metric and neuropsychological score, and whole brain atrophy, using the BBSI, to detect a 20, 30, 40, and 50% reduction in yearly change.

## Results

### Demographics, Neuropsychological Performance, and Changes in Whole Brain Volume

Demographic and volumetric imaging characteristics of study participants are shown in Table1; of the 23 bvFTD patients, 9 had apparently sporadic bvFTD, having no family history of bvFTD and a negative test for relevant genetic mutations; 8 patients had mutations in *MAPT* (5 exon 10 +16 mutations, 2 R407W mutations, and 1 P301L mutation); 4 patients had a *C9ORF72* mutation. Patients and cognitively normal participants were matched for age, gender, and total intracranial volume. Compared with cognitively normal participants, those with bvFTD had significantly lower Mini-Mental State Examination scores (*p* < 0.01) and whole brain volumes (*p* ≤ 0.001) at baseline and follow-up. Rates of atrophy were greatest (*p* = 0.002) in the bvFTD group, with the highest rate of volume loss occurring in those with *MAPT* mutations (*p* = 0.001).

**Table 1:** Study Participants' Clinical and Imaging Characteristics

Characteristic	Controls, n = 18		*MAPT*, n = 8		*C9ORF72*, n = 4		Sporadic, n = 11		All bvFTD, n = 23		*p*^a^
	Mean	SD	Mean	SD	Mean	SD	Mean	SD	Mean	SD	
Age at baseline, yr	61.3	9.5	56.7	8.9	64.1	8.7	68.8	8.4	63.8	10.0	0.4
Disease duration at baseline, yr			5.2	5.4	9.3	5.9	6.8	4.7	6.7	5.1	N/A
Sex, M/F, No.	12/6		5/3		4/0		10/1		18/5		0.7^b^
Interscan interval, yr	1.4	0.5	1.3	0.5	1.0	0.1	1.2	0.5	1.2	0.4	0.2
Education, yr	16.55	1.42	14.6	3.9	15.5	4.1	16.6	3.0	15.5	3.5	0.3
MMSE baseline	29.7	0.6	25.5	4.2	2.5	6.9	25.8	3.4	25.3	4.2	<0.001^c^
MMSE follow-up	29.7	0.5	26.3	5.4	25.3	4.3	25.1	3.5	25.6	4.2	0.002^c^
TIV, ml	1,572	134	1,503	135	1,649	135	1,565	123	1,556	137	0.6
Whole brain volume, baseline, ml	1,193	91	1,047	88	1,192	93	1,026	48	1,070	95	0.001
Whole brain volume, follow-up, ml	1,184	95	1,028	95	1,167	93	1,001	37	1,047	95	<0.001
BBSI, ml/yr	5.2	6.7	15.7	6.7	14.4	17.8	14.8	10.6	15.2	10.4	0.002

a Linear regression comparing controls with all bvFTD subjects.

b Fisher exact test.

c Wilcoxon rank sum test.

BBSI = brain boundary shift integral; bvFTD = behavioral variant frontotemporal dementia; *C9ORF72* = chromosome 9 open-reading frame 72; F = female; M = male; *MAPT* = microtubule-associated protein tau; MMSE = Mini-Mental State Examination; SD = standard deviation; TIV = total intracranial volume.

Neuropsychological performance at baseline and longitudinally is shown in Table2 (see Supplementary Table1 for bvFTD subgroups). Compared with cognitively normal participants, at baseline, those with bvFTD had significantly poorer performance on tests of general intellect, recognition memory, naming, object perception, executive function, emotion recognition, and social inference. Longitudinally, the greatest change observed in the bvFTD group was a 30.4% decline in score on the graded naming test compared with a 2% increase in cognitively normal participants (*p* < 0.001).

**Table 2:** Study Participants' Neuropsychological Performance at Baseline and Follow-up

Measure	Control, n = 18					bvFTD, n = 23					*p*, Baseline	*p*, Change
	Baseline		Change Over Time from Baseline		Baseline		Change Over Time from Baseline					
	Raw Score	SD	%/yr	95% CI		Raw Score	SD	%/yr	95% CI			
WASI												
VIQ	122.1	9.4	−2.6	−6.2	1.0	87.3	22.1	0.1	−5.0	5.3	<0.001	0.96
PIQ	120.1	9.2	−1.4	−4.6	1.9	89.9	19.3	4.0	−0.7	8.7	<0.001	0.09
Recognition memory test												
Words (/50)	47.9	2.6	0.7	−4.1	5.5	33.3	6.6	0.0	−7.0	7.0	<0.001	1.00
Faces (/50)	43.4	4.6	2.3	−2.7	7.3	36.0	8.3	−5.5	−12.7	1.8	<0.001	0.14
Graded naming test (/30)	26.4	2.2	2.0	−9.2	13.2	11.6	8.5	−30.4	−47.2	−13.6	<0.001	<0.001
Graded arithmetic test (/24)	14.7	4.5	−3.1	−11.6	5.4	13.0	6.4	−8.9	−21.0	3.1	0.24	0.15
Visual object space perception (/20)	19.1	1.0	−2.3	−11.0	6.3	16.0	3.8	6.0	−6.6	18.5	0.002	0.35
Executive function												
DKEFS color naming (max 90 seconds)	30.2	3.2	0.9	−7.0	8.8	42.3	20.3	5.4	−6.0	16.8	0.01	0.35
DKEFS ink color naming (max 180 seconds)	55.7	11.6	0.1	−6.3	6.5	88.8	37.6	8.1	−1.1	17.3	<0.001	0.08
TASIT^a^												
Emotion recognition (/14)	11.4	1.2	−1.2	−10.7	8.4	8.0	1.9	−3.7	−17.3	9.9	<0.001	0.59
Social inference task (/36)	32.1	2.9	2.0	−3.9	7.9	22.5	4.8	−5.1	−13.4	3.3	<0.001	0.23

Scores shown are raw scores for each test with maximum scores shown in parentheses. Probability values denote significance level comparing behavioral scores (raw score at baseline and change from baseline over time) of all bvFTD participants with control participants after adjustment for age, gender, and disease duration.

a Total scores on the TASIT are scaled scores.

vFTD = behavioral variant frontotemporal dementia; CI = confidence interval; DKEFS = Delis–Kaplan Executive Frontal System; PIQ = performance intelligence quotient; SD = standard deviation; TASIT = Awareness of Social Inference Test; VIQ = verbal intelligence quotient; WASI = Wechsler Abbreviated Scale of Intelligence.

### Cross-Sectional DTI Results

Cross-sectional DTI metric data for FA and MD are shown in Table3 (see Supplementary Table2 for RD and AX). At baseline, compared with cognitively normal participants, bvFTD patients as a group had significantly lower (*p* ≤ 0.02) FA and increased MD within the body of the corpus callosum, bilateral uncinate fasciculus, and right parahippocampal cingulum, and additionally increased MD in left parahippocampal cingulum. In *MAPT* mutation carriers, FA was 6.4% (95% confidence interval [CI] = −11 to −2%; *p* < 0.009) lower in right parahippocampal cingulum compared with controls, whereas MD was 17.7% higher in right uncinate fasciculus (95% CI = 9.0 to 27%; *p* < 0.001) and 9.4% higher in right parahippocampal cingulum (95% CI = 2.1 to 16.6%, *p* = 0.01). In sporadic bvFTD, FA was 10.3% lower in left uncinate fasciculus (95% CI = −18.2 to −2.5%; *p* = 0.01) and 6.7% lower in right parahippocampal cingulum (95% CI = −12.7 to −0.6%; *p* = 0.03) compared with controls, whereas MD was 31% higher in left uncinate fasciculus (95% CI = 13.5 to 50%; *p* = 0.002) and 15% higher in right parahippocampal cingulum (95% CI = 6.1 to 24%, *p* = 0.002). In *C9ORF72* mutation carriers, FA was 11% lower in both right and left superior cerebellar peduncle (right: 95% CI = −21.1 to −1.0%, *p* = 0.03; left: 95% CI = −20.1 to −1.9%, *p* = 0.02) compared with controls.

**Table 3:** Baseline Diffusion Tensor Imaging Metric Data for Individual White Matter Regions of Interest for Control Participants and Patients

Regions of Interest	Controls, n = 18		bvFTD, n = 19		% Difference	95% Confidence Interval		*p*^a^
	Mean	SD	Mean	SD				
FA								
Genu corpus callosum	0.74	0.03	0.73	0.04	0.34	−2.64	3.31	0.8
Body corpus callosum	0.70	0.04	0.66	0.04	−4.87	−8.68	−1.07	0.01
Splenium corpus callosum	0.79	0.02	0.78	0.02	−1.27	−3.15	0.61	0.2
Cingulum (paracallosal) R	0.63	0.05	0.58	0.06	−4.26	−9.25	0.73	0.09
Cingulum (paracallosal) L	0.60	0.04	0.55	0.07	−2.25	−7.08	2.59	0.4
Cingulum (parahippocampal) R	0.43	0.04	0.38	0.04	−5.77	−9.59	−1.96	0.004
Cingulum (parahippocampal) L	0.45	0.04	0.40	0.04	−5.26	−9.19	−1.33	0.01
Fornix	0.59	0.03	0.57	0.04	−1.91	−4.21	0.40	0.1
Uncinate fasciculus R	0.48	0.05	0.43	0.04	−4.65	−8.62	−0.67	0.02
Uncinate fasciculus L	0.48	0.04	0.43	0.07	−6.24	−11.32	−1.16	0.02
Corticospinal tract R	0.62	0.05	0.62	0.07	−1.45	−7.15	4.25	0.6
Corticospinal tract L	0.65	0.04	0.64	0.05	−2.15	−6.12	1.81	0.3
SCP R	0.79	0.05	0.78	0.06	−2.24	−7.21	2.72	0.4
SCP L	0.79	0.04	0.77	0.05	−2.88	−7.19	1.42	0.2
MD, 10^−3^mm^2^/s								
Genu corpus callosum	0.76	0.04	0.80	0.08	0.29	−4.81	5.40	0.9
Body corpus callosum	0.80	0.06	0.88	0.08	8.66	2.55	14.76	0.01
Splenium corpus callosum	0.74	0.03	0.77	0.05	3.80	−0.12	7.72	0.06
Cingulum (paracallosal) R	0.72	0.04	0.72	0.05	1.64	−2.48	5.76	0.4
Cingulum (paracallosal) L	0.71	0.04	0.73	0.05	0.47	−3.49	4.43	0.8
Cingulum (parahippocampal) R	0.74	0.03	0.85	0.12	9.32	1.49	17.15	0.02
Cingulum (parahippocampal) L	0.73	0.04	0.83	0.11	10.63	3.09	18.17	0.007
Fornix	0.84	0.06	0.91	0.10	5.44	−0.09	10.97	0.05
Uncinate fasciculus R	0.70	0.04	0.83	0.13	13.50	4.81	22.19	0.003
Uncinate fasciculus L	0.71	0.03	0.85	0.18	14.87	2.65	27.10	0.02
Corticospinal tract R	0.66	0.06	0.67	0.09	1.84	−5.28	8.96	0.6
Corticospinal tract L	0.62	0.05	0.62	0.09	2.45	−4.32	9.22	0.5
SCP R	0.85	0.07	0.87	0.09	5.41	−1.96	12.78	0.1
SCP L	0.78	0.07	0.80	0.07	4.22	−2.29	10.72	0.2

a Linear regression comparing bvFTD with controls after adjusting for age, gender, and disease duration.

vFTD = behavioral variant frontotemporal dementia; FA = fractional anisotropy; L = left; MD = mean diffusivity; R = right; SCP = superior cerebellar peduncle; SD = standard deviation.

### Longitudinal DTI Changes in bvFTD

**Figure 2:**
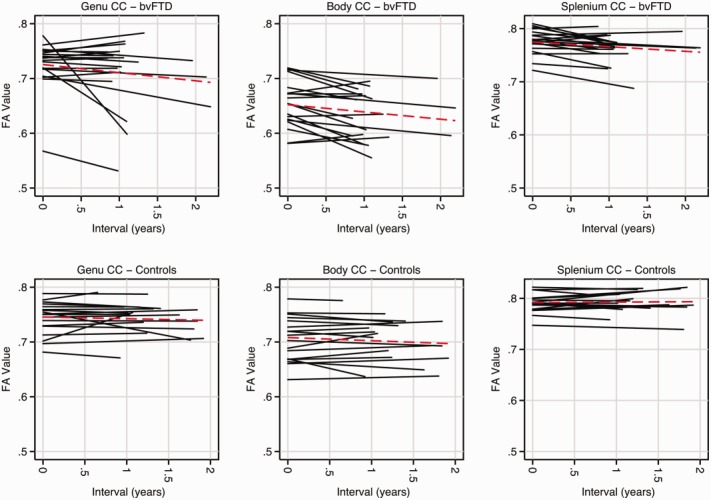
Plots of each participant's fractional anisotropy (FA) over time within subdivisions of the corpus callosum (CC). Each line represents a single subject, with behavioral variant frontotemporal dementia (bvFTD) participants across the top and controls along the bottom. Red dashed lines indicate the mean trajectory. [Color figure can be viewed in the online issue, which is available at http://www.annalsofneurology.org.]

**Figure 3:**
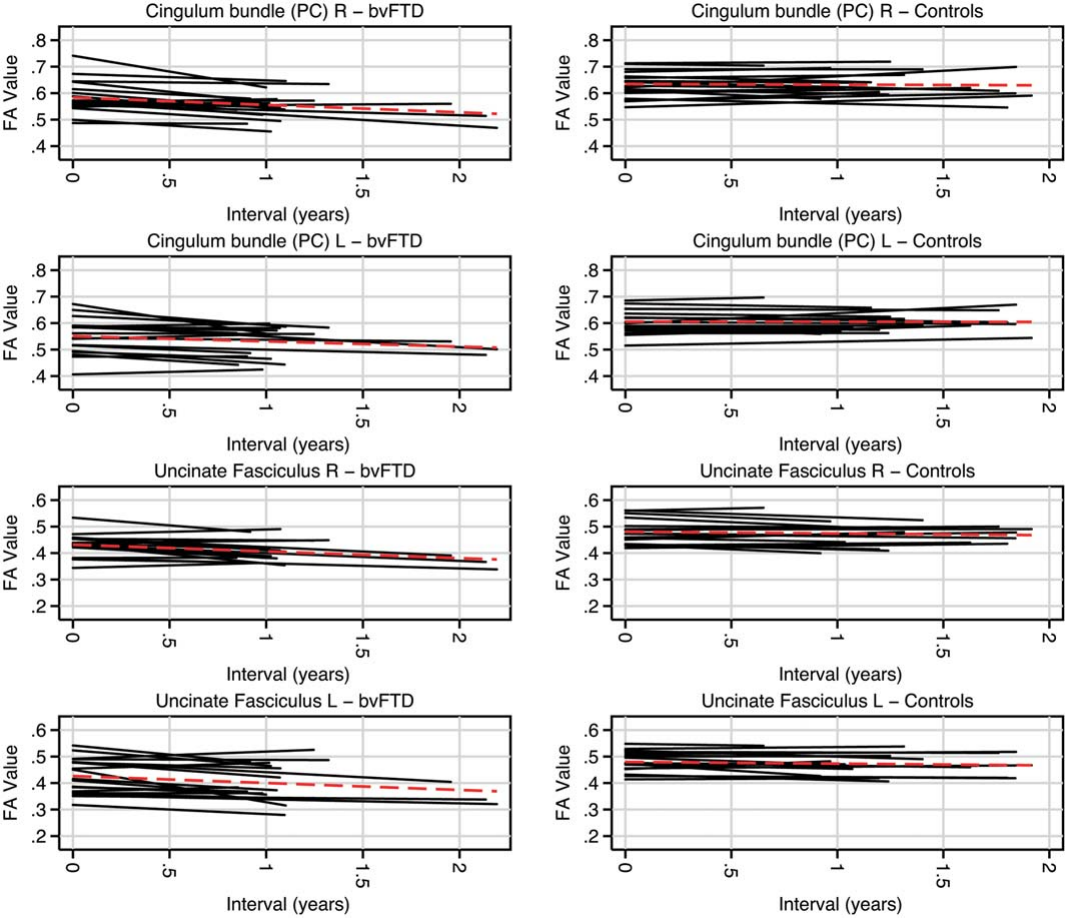
Plots of each participant's fractional anisotropy (FA) over time within the uncinate fasciculus and paracallosal (PC) cingulum bundle. Each line represents a single subject, with behavioral variant frontotemporal dementia (bvFTD) participants along the left and controls along the right. Red dashed lines indicate the mean trajectory. L = left; R = right. [Color figure can be viewed in the online issue, which is available at http://www.annalsofneurology.org.]

Rates of change for each region of interest and diffusivity metric are shown in Table4 and Figures 2 and 3 (see Supplementary Table3 for RD and AX). Longitudinally, compared with cognitively normal participants, bvFTD patients as a group had the largest reductions in FA within bilateral paracallosal cingulum (right: −6.8%/yr, 95% CI = −8.0 to −2.7%, *p* < 0.001; left, −5.5%/yr, 95% CI = −6.9 to −2.2%, *p* < 0.001) and bilateral uncinate fasciculus (right, −4.2%/yr, 95% CI = −8.7 to −2.7%, *p* < 0.001; left: −3.1%/yr, 95% CI = −8.6 to −1.5%, *p* = 0.005). The largest increases in MD were within bilateral uncinate fasciculus (right: 5.1%/yr, 95% CI = 2.3 to 8.0%, *p* < 0.001; left: 6.2%/yr, 95% CI = 1.6 to 10.8%, *p* = 0.01;) and bilateral parahippocampal cingulum (right: 4.3%/yr, 95% CI = 1.6 to 7.1%, *p* = 0.002; left, 5.0%/yr, 95% CI = 1.1 to 9.0%, *p* = 0.01).

**Table 4:** Estimated Percentage per Year Difference in the Rate of Change of FA and MD for bvFTD Patients and bvFTD Subgroups, by Region, Compared with Controls

Region of Interest	bvFTD, n = 19				bvFTD, *MAPT*, n = 8				bvFTD, Sporadic, n = 7				bvFTD, *C9ORF72*, n = 4			
	%/yr Change	95% CI		*p*	%/yr Change	95% CI		*p*	%/yr Change	95% CI		*p*	%/yr Change	95% CI		*p*
FA																
Genu CC	−2.0	−4.7	0.3	0.09	−2.0	−5.2	1.2	0.22	−3.1	−5.5	−0.8	0.01	0.5	−1.9	2.9	0.68
Body CC	−2.4	−4.0	−0.8	0.003	−3.3	−4.8	−1.8	<0.001	−1.5	−3.4	0.4	0.12	−2.1	−4.7	0.6	0.13
Splenium CC	−2.1	−2.4	−0.7	0.001	−1.4	−2.4	−0.4	0.004	−1.4	−2.4	−0.4	0.005	−2.1	−3.8	−0.3	0.02
Cingulum (paracallosal) R	−6.8	−8.0	−2.7	<0.001	−4.0	−7.2	−0.8	0.01	−6.7	−10.0	−3.4	<0.001	−6.8	−11.3	−2.2	0.004
Cingulum (paracallosal) L	−5.5	−6.9	−2.2	<0.001	−4.6	−7.4	−1.8	0.001	−5.6	−8.3	−2.8	<0.001	−1.7	−5.6	2.1	0.38
Cingulum (parahippocampal) R	3.5	−3.4	2.7	0.82	−1.5	−5.2	2.2	0.42	1.9	−1.9	5.7	0.98	−2.7	−8.9	3.5	0.39
Cingulum (parahippocampal) L	−1.8	−7.7	0.9	0.12	−6.5	−11.2	−1.8	0.007	0.1	−5.4	5.6	0.01	−2.4	−11.4	6.6	0.60
Fornix	−1.2	−4.1	0.3	0.09	−1.8	−4.6	0.9	0.19	−2.7	−5.0	−0.5	0.02	0.8	−1.2	2.8	0.45
Uncinate fasciculus R	−4.2	−8.1	−2.7	<0.001	−7.2	−9.7	−4.7	<0.001	−3.5	−7.2	0.2	0.07	−4.3	−9.1	0.5	0.08
Uncinate fasciculus L	−3.1	−8.6	−1.5	0.005	−7.9	−12.0	−3.7	<0.001	−1.5	−4.6	1.6	0.34	−4.8	−9.6	−0.1	0.05
Corticospinal tract R	−2.9	−8.6	0.3	0.07	−3.4	−8.5	1.7	0.19	−6.6	−12.5	−0.7	0.03	0.5	−7.5	8.4	0.91
Corticospinal tract L	−1.8	−8.2	0.1	0.06	−4.0	−9.1	1.0	0.12	−3.2	−8.6	2.3	0.25	−7.8	−16.9	1.2	0.09
SCP R	0.4	−5.0	1.3	0.25	−1.2	−4.5	2.1	0.48	−1.2	−4.5	2.1	0.48	−6.8	−13.8	0.3	0.06
SCP L	−0.5	−5.1	0.9	0.18	−2.6	−5.6	0.4	0.09	−1.8	−5.0	1.4	0.09	−1.3	−8.9	6.3	0.74
MD, 10^−3^mm^2^/s																
Genu CC	2.7	−1.1	6.6	0.16	2.5	−2.8	7.8	0.36	3.6	0.4	6.8	0.03	−0.7	−4.8	3.4	0.73
Body CC	2.5	0.8	4.3	0.003	3.1	1.3	4.9	0.001	1.4	−0.9	3.8	0.23	3.6	0.7	6.6	0.01
Splenium CC	2.7	0.9	4.5	0.003	2.8	0.5	5.1	0.02	2.4	0.4	4.4	0.02	3.2	−0.4	6.7	0.08
Cingulum (paracallosal) R	2.9	0.8	4.9	0.01	2.3	−0.4	5	0.09	3.8	1.5	6	0.001	2.0	−1.8	5.7	0.30
Cingulum (paracallosal) L	2.7	0.6	4.8	0.01	2.3	−0.3	4.9	0.08	3.4	0.8	6.1	0.01	1.9	−1.7	5.5	0.29
Cingulum (parahippocampal) R	4.3	1.6	7.1	0.002	5.6	2.7	8.6	<0.001	3.3	−0.2	6.8	0.06	4.5	−1.8	10.7	0.16
Cingulum (parahippocampal) L	5.0	1.1	9	0.01	8.3	4.3	12.2	<0.001	1.8	−2.8	6.3	0.45	2.8	−4.4	10.1	0.44
Fornix	1.8	−1.1	4.8	0.22	1.8	−2.1	5.7	0.38	2.4	−0.5	5.2	0.11	−0.9	−4.2	2.4	0.59
Uncinate fasciculus R	5.1	2.3	8	<0.001	6.7	3.7	9.6	<0.001	4.0	0.7	7.4	0.02	3.0	−2.6	8.6	0.30
Uncinate fasciculus L	6.2	1.6	10.8	0.01	10.9	5.2	16.5	<0.001	0.8	−1.7	3.3	0.52	6.0	0.8	11.2	0.02
Corticospinal tract R	2.2	−3.2	7.6	0.43	3.2	−3.9	10.2	0.38	2.8	−3.9	9.6	0.41	−1.1	−12.1	9.9	0.84
Corticospinal tract L	3.9	−1.5	9.4	0.16	4.3	−2.9	11.5	0.24	2.0	−4.7	8.6	0.56	9.3	−2.4	21	0.12
SCP R	2.2	−3.2	7.6	0.43	3.2	−3.9	10.2	0.38	2.8	−3.9	9.6	0.41	−1.1	−12.1	9.9	0.84
SCP L	5.1	0.7	9.5	0.02	5.6	0.7	10.5	0.02	4.0	−1.0	9.0	0.12	7.4	−2.5	17.2	0.14

bvFTD = behavioral variant frontotemporal dementia; *C9ORF72* = chromosome 9 open-reading frame 72; CC = corpus callosum; CI = confidence interval; FA = fractional anisotropy; L = left; *MAPT* = microtubule-associated protein tau; MD = mean diffusivity; R = right; SCP = superior cerebellar peduncle.

### Longitudinal DTI Changes in bvFTD Subgroups

Rates of change for each region of interest and diffusivity metric are shown in Table4 (see Supplementary Table3 for RD and AX). Compared with cognitively normal participants, the largest reductions in FA were within bilateral uncinate fasciculus (right: −7.2%/yr, 95% CI = −9.7 to −4.7%, *p* < 0.001; left: −7.9%/yr, 95% CI = −12.0 to −3.7%, *p* < 0.001) in *MAPT* mutation carriers; bilateral paracallosal cingulum bundle (right: −6.7%/yr, 95% CI = −10.0 to −3.4%, *p* < 0.001; left: −5.6%/yr, 95% CI = −8.3 to −2.8%, *p* < 0.001) in those with sporadic bvFTD, and in right paracallosal cingulum bundle (6.8%/yr, 95% CI = −11.3 to 2.2%, *p* = 0.004) in *C9ORF72* mutation carriers. Compared with cognitively normal participants, the largest increases in MD were in bilateral uncinate fasciculus (right: 6.7%/yr, 95% CI = 3.7 to 9.6%, *p* < 0.001; left: 10.9%/yr, 95% CI = 5.2 to 16.5%, *p* < 0.001) in *MAPT* mutation carriers, bilateral paracallosal cingulum bundle (right: 3.8%/yr, 95% CI = 1.5 to 6.0%, *p* = 0.001; left: 3.4%/yr, 95% CI = 0.8 to 6.1%, *p* = 0.01) in sporadic bvFTD, and left uncinate fasciculus (6.0%/yr, 95% CI = 0.8 to 11.2%, *p* = 0.02) in *C9ORF72* mutation carriers.

### Cross-Sectional and Longitudinal DTI Metric Sensitivity and Specificity

AUC data indicated that classification of control and bvFTD groups cross-sectionally were best achieved using RD, measured within the right uncinate fasciculus (AUC = 0.86, specificity = 94%, sensitivity = 68%), followed by MD (AUC = 0.83, specificity = 89%, sensitivity = 74%), FA (AUC = 0.79, specificity = 67%, sensitivity = 84%), and AX (AUC = 0.75, specificity = 83%, sensitivity = 74%). Classification of control and bvFTD groups longitudinally were best achieved using FA change, measured within the right cingulum bundle (AUC = 0.79, specificity = 94%, sensitivity = 63%), followed by MD (AUC = 0.77, specificity = 89%, sensitivity = 68%), and RD (AUC = 0.76, specificity = 100%, sensitivity = 58%), with AX performing less favorably (AUC = 0.53, specificity = 67%, sensitivity = 47%).

### Sample Size Estimations

Sample sizes required for future clinical trials were calculated using annualized change score in 3 potential outcome measures: whole brain atrophy (using BBSI), change in graded naming test, and DTI change within either right paracallosal cingulum or right uncinate fasciculus (chosen on the basis of statistical significance). Sample size estimates corrected for control rates of change are displayed in Table5. Sample size estimates based on changes in FA were smaller than sample size estimates based on other outcome measures.

**Table 5:** Sample Size Estimates^a^ per Treatment Arm of a Clinical Trial Comparing 3 Different Outcome Measures to Detect a 20 to 50% Reduction in Rates of Change

	DTI Change, %/yr				BBSI, ml/yr	Change in Graded Naming Test
	Right Cingulum Bundle		Right Uncinate Fasciculus			
% Change	FA	MD	RD	AX		
20	276	1,031	531	1,229	507	1,524
30	123	459	236	546	226	685
40	69	258	133	308	127	381
50	45	165	85	197	82	246

Sample size estimates have been adjusted for control rates of change.

a β = 80%, α = 0.05.

AX = axial diffusivity; BBSI = brain boundary shift integral (a measure of whole brain atrophy); DTI = diffusion tensor imaging; FA = fractional anisotropy; MD = mean diffusivity; RD = radial diffusivity.

## Discussion

This study is among the first to demonstrate the feasibility of carrying out longitudinal DTI in patients with bvFTD. Compared with previous DTI studies, we used what we believe to be an improved approach for DTI spatial normalization, which enforces longitudinally and cross-sectionally consistent and accurate region of interest segmentations, thus reducing potential noise within the DTI data set, which is often a significant methodological problem. Using these improved DTI methods, we (1) report both core and mutation-specific patterns of white matter change in bvFTD; and (2) demonstrate that longitudinal DTI is a feasible outcome measure for clinical trials, requiring smaller sample sizes than other more conventional outcome measures.

We found that decreasing FA and increasing MD within bilateral paracallosal cingulum bundle, body of the corpus callosum, and bilateral uncinate fasciculus was the most consistent finding across all bvFTD groups. These findings are in line with a number of cross-sectional DTI studies in bvFTD.^18^–^22^ In terms of biological validity, the cingulum bundle is a key association tract linking anterior cingulate and prefrontal cortices and likely underpins executive processes. It is of interest that the greatest cross-sectional differences in the cingulum were within the parahippocampal subdivision. This suggests this ventral subdivision of the cingulum, linking structures within the limbic system such as hippocampus and posterior cingulate cortex, is involved early in the disease process, and may account for some of the more subtle changes in general intellectual function seen in early bvFTD.^23^ However, over time the paracallosal cingulum, linking cingulate and prefrontal cortices and overlapping with the functionally relevant Salience Network,^10^ showed greater disease progression. These longitudinal imaging changes may be associated with the clinical evolution of bvFTD, with progressive disintegration of social cognition and executive skills such as response inhibition and set shifting.^24^,^25^ In addition, alterations to the cingulum bundle have been found in Alzheimer disease^26^ and several psychiatric disorders.^27^ The corpus callosum is the major commissure integrating right and left hemispheric cognitive processes. Damage to the corpus callosum has been linked to a range of abnormal social behaviours^28^ and may lead to disconnection between brain regions that integrate semantic knowledge (left hemisphere) with emotional meaning (right hemisphere), impacting on the ability to interpret paralinguistic information and situational context, a common feature in bvFTD.^29^ The uncinate fasciculus has been implicated in cross-sectional studies^19^,^21^,^22^ and is an important tract connecting orbitofrontal cortex and anterior temporal lobes.

Different patterns of white matter change were associated with different FTD subtypes. In *MAPT* mutation carriers, the most robust changes were within bilateral uncinate fasciculus, bilateral paracallosal and parahippocampal cingulum, and the body of the corpus callosum; in the *C9ORF72* mutation carriers, within corpus callosum and right paracallosal cingulum bundle; and in sporadic bvFTD, within the bilateral paracallosal cingulum bundle. *MAPT* mutation carriers were the only group to have significant change over time within both right and left uncinate fasciculus, with a high burden of white matter change occurring in medial temporal lobe regions. Breakdown of these tracts is biologically plausible, given that these tracts link gray matter structures that show preferentially more atrophy in affected *MAPT* mutation carriers.^30^ Uncinate fasciculus pathology has also been demonstrated in presymptomatic *MAPT* mutation carriers.^2^ Furthermore, breakdown of these tracts may account for deficits in cognitive processes such as episodic memory, semantic knowledge, and emotion processing seen in *MAPT* mutation carriers.^23^,^31^ Although interpretation of change within the *C9ORF72* group must be viewed in terms of the small sample size, it is of interest that only this group showed significant difference in bilateral superior cerebellar peduncles cross-sectionally and neared significance (*p* = 0.06) longitudinally. This might be surprising for a “frontotemporal” dementia; however, this finding is consistent with other data suggesting that changes within the cerebellum and associated structures may be a particular hallmark of *C9ORF72* mutation carriers.^5^,^32^,^33^ In the sporadic bvFTD group, bilateral paracallosal cingulum was the primary region of interest affected, which is in keeping with the broader group level finding. The heterogeneity of the group could provide a possible explanation for the emergence of a more limited profile of white matter change in this group. However, it is noteworthy that the paracallosal cingulum displayed significant differences from controls across subgroups both cross-sectionally and longitudinally. This suggests that damage to these fibers is common across the spectrum of bvFTD, making it a potentially appealing tract to study longitudinally.

An important issue that remains poorly understood is the choice of DTI metric to detect and track white matter change. To date, most longitudinal DTI studies have reported only changes in FA,^34^,^35^ although more recently studies have also reported on changes in mean diffusivity.^14^,^36^ The current study compared the performance of a range of DTI metrics and suggests that the optimal region of interest and DTI metric may differ cross-sectionally and longitudinally. From the cross-sectional data, RD within the right uncinate fasciculus performed best, whereas FA change within the right paracallosal cingulum bundle performed best longitudinally and yielded the lowest sample size estimation. These differences may in part be explained by variations in disease neurobiology and tract anatomy. Changes in RD and AX are thought to reflect local changes in myelination and axonal damage, respectively, whereas FA, a composite of these measures, reflects changes in the overall direction of diffusion reflecting more general white matter integrity. RD and AX may have superior ability to detect focal white matter pathology, making them sensitive cross-sectional metrics. The longitudinal changes detected may reflect Wallerian degeneration, a process commonly seen in a range of neurodegenerative disorders,^37^ which may be better detected by FA.^38^ However, we do not argue that FA is the only metric to capture change, particularly as FA values are determined to some degree by changes in RD. The performance of particular DTI metrics is likely affected by individual patient variability, particularly relevant to bvFTD given its pathological heterogeneity, which may result in variable trajectories of disease progression (see Figs 2 and 3). It is possible that certain white matter structures are more suited to longitudinal measurements than others, perhaps due to their orientation or size, thus allowing better registration. For example, change within the genu and body of the corpus callosum appeared more stable in control participants, whereas in other structures the trajectory of change was more variable (see Figs 2 and 3).

Understanding how disease neurobiology results in specific changes to DTI metrics will require further studies with larger pathologically confirmed cohorts, including the progranulin genetic subtype, which the current study lacks. Larger studies will be required to confirm these data as well as to examine changes within other white matter structures not included here. Further improvements to image acquisition and analysis through the use of multishell acquisitions may improve spatial resolution of the data and provide greater information on white matter integrity.^39^

We have demonstrated that a within-subject measure of DTI change is a potentially useful disease biomarker with the ability to detect greater differences across white matter regions compared with cross-sectional measures. This, coupled with lower sample size requirements, suggests that longitudinal DTI may be an important biomarker for disease monitoring with particular implications for future clinical trials.
