# Supplementary material for: Longitudinal Diffusion Tensor Imaging in Frontotemporal Dementia
Source: Ann Neurol. 2014 Nov 17;77(1):33–46. doi: 10.1002/ana.24296 (PMC4305215; doi:10.1002/ana.24296)
Supplement: Supplementary file 1 [file ana0077-0033-sd1.docx]

|  | **Sporadic (n=11)** | | | | | | | **MAPT (n=8)** | | | | | | | **C9ORF72 (n=4)** | | | | | | |
| --- | --- | --- | --- | --- | --- | --- | --- | --- | --- | --- | --- | --- | --- | --- | --- | --- | --- | --- | --- | --- | --- |
|  | Baseline | | Change over time from baseline | | | **p-value baseline** | **p-value change** | Baseline | | Change over time from baseline | | | **p-value baseline** | **p-value change** | Baseline | | Change over time from baseline | | | **p-value baseline** | **p-value change** |
|  | Raw score | SD | %/year | 95% CI | |  |  | Raw score | SD | %/year | 95% CI | |  |  | Raw score | SD | %/year | 95% CI | |  |  |
| **WASI** |  |  |  |  |  |  |  |  |  |  |  |  |  |  |  |  |  |  |  |  |  |
| VIQ | 95.3 | 17.2 | -3.8 | -9.5 | 2.0 | <0.001 | 0.2 | 80.6 | 25.7 | 2.1 | -2.3 | 6.5 | <0.001 | 0.4 | 78.8 | 24.8 | 17.0 | 9.1 | 25.0 | <0.001 | <0.001 |
| PIQ | 91.2 | 17.8 | 0.1 | -4.6 | 4.9 | <0.001 | 1.0 | 91.1 | 18.3 | 6.4 | 2.1 | 10.8 | <0.001 | 0.004 | 83.8 | 28.9 | 16.5 | 8.8 | 24.3 | <0.001 | <0.001 |
| **Recognition Memory Test** | | | |  |  |  |  |  |  |  |  |  |  |  |  |  |  |  |  |  |  |
| Words (/50) | 33.1 | 6.8 | -9.1 | -14.2 | -4.1 | <0.001 | <0.001 | 33.9 | 6.8 | -6.0 | -15.9 | 3.9 | <0.001 | 0.2 | 35.5 | 7.4 | 19.0 | 7.6 | 30.5 | <0.001 | <0.001 |
| Faces (/50) | 37.6 | 8.4 | -2.3 | -10.8 | 6.1 | <0.001 | 0.6 | 34.0 | 9.1 | 3.7 | -1.8 | 9.2 | <0.001 | 0.2 | 32.8 | 7.6 | 1.9 | -4.9 | 8.7 | <0.001 | 0.6 |
| **Naming (/30)** | 12.6 | 7.8 | -45.3 | -62.2 | -28.5 | <0.001 | <0.001 | 6.6 | 7.8 | -18.5 | -36.4 | -0.7 | <0.001 | 0.04 | 18.8 | 6.7 | 0.6 | -12.8 | 13.9 | <0.001 | 0.9 |
| **Arithematic (/24)** | 14.4 | 6.3 | -10.9 | -22.2 | 0.4 | 0.2 | 0.1 | 13.1 | 4.5 | -12.4 | -29.5 | 4.7 | 0.5 | 0.2 | 8.8 | 9.4 | 16.4 | -10.2 | 43.0 | 0.1 | 0.2 |
| **VOSP (/20)** | 16.4 | 4.3 | 3.7 | -11.1 | 18.6 | 0.02 | 0.6 | 16.1 | 2.0 | 2.0 | -4.8 | 8.8 | <0.001 | 0.6 | 15.0 | 5.7 | 27.5 | 5.4 | 49.7 | <0.001 | 0.02 |
| **Executive Function** | |  |  |  |  |  |  |  |  |  |  |  |  |  |  |  |  |  |  |  |  |
| DKEFS Colour Naming (max 90 secs) | 46.3 | 22.5 | 11.5 | 2.8 | 20.2 | 0.002 | 0.01 | 31.5 | 7.0 | 9.9 | -0.1 | 20.0 | 0.4 | 0.1 | 53.0 | 26.4 | -35.7 | -61.1 | -10.3 | <0.001 | 0.01 |
| DKEFS Ink Colour Naming (max 180 secs) | 91.9 | 38.4 | 10.8 | 2.2 | 19.3 | <0.001 | 0.01 | 71.6 | 20.9 | 9.1 | -1.7 | 20.0 | 0.0 | 0.1 | 114.5 | 52.3 | -5.8 | -23.9 | 12.2 | <0.001 | 0.5 |
| **TASIT** |  |  |  |  |  |  |  |  |  |  |  |  |  |  |  |  |  |  |  |  |  |
| Emotion Recognition (/14) | 7.3 | 1.8 | -3.6 | -20.9 | 13.7 | <0.001 | 0.7 | 8.7 | 2.3 | -6.5 | -17.6 | 4.6 | <0.001 | 0.3 | 8.7 | 1.2 | 9.6 | -9.8 | 29.0 | <0.001 | 0.3 |
| Social Inference Task (/36) | 21.4 | 4.7 | -10.7 | -18.8 | -2.5 | <0.001 | 0.01 | 23.3 | 5.9 | 4.0 | -6.2 | 14.2 | <0.001 | 0.4 | 24.0 | 3.6 | 0.7 | -7.8 | 9.1 | <0.001 | 0.9 |

**Supplementary Table 1.** bvFTD subgroup neuropsychological performance at baseline and follow up. Scores shown are raw scores for each test with maximum scores shown in parentheses. p-values denote significance level comparing behavioural scores (raw score at baseline and change from baseline over time) of bvFTD subgroups with control participants after adjustment for age, gender and disease duration. ^ Total scores on the TASIT are scaled scores. CI, Confidence Interval, DKEFS, Delis-Kaplan Executive Frontal System; SD, standard deviation; TASIT, The Awareness of Social Inference Test

|  | **Controls (n=18)** | | **bvFTD (n=19)** | | % Difference | 95% Confidence Interval | | p-value* |
| --- | --- | --- | --- | --- | --- | --- | --- | --- |
| ***Regions of interest (RD 10^-3^mm ^2^/s)*** | Mean | SD | Mean | SD |  |  |  |  |
|  |  |  |  |  |  |  |  |  |
| Genu Corpus Callosum | 0.34 | 0.05 | 0.38 | 0.09 | -0.27 | -5.73 | 5.18 | 0.92 |
| Body Corpus Callosum | 0.39 | 0.06 | 0.48 | 0.09 | 9.29 | 2.52 | 16.06 | 0.01 |
| Splenium Corpus Callosum | 0.30 | 0.03 | 0.33 | 0.05 | 3.05 | -0.54 | 6.65 | 0.09 |
| Cingulum (paracallosal) R | 0.41 | 0.05 | 0.45 | 0.06 | 3.90 | -1.48 | 9.27 | 0.15 |
| Cingulum (paracallosal) L | 0.43 | 0.05 | 0.48 | 0.07 | 1.93 | -3.22 | 7.09 | 0.45 |
| Cingulum (parahippocampal) R | 0.55 | 0.04 | 0.68 | 0.12 | 10.63 | 2.30 | 18.96 | 0.01 |
| Cingulum (parahippocampal) L | 0.54 | 0.05 | 0.65 | 0.11 | 11.15 | 3.18 | 19.12 | 0.01 |
| Fornix | 0.51 | 0.06 | 0.59 | 0.10 | 5.35 | -0.37 | 11.07 | 0.07 |
| Uncinate Fasciculus R | 0.50 | 0.05 | 0.64 | 0.12 | 14.73 | 6.14 | 23.32 | 0.00 |
| Uncinate Fasciculus L | 0.51 | 0.04 | 0.66 | 0.18 | 16.63 | 4.35 | 28.91 | 0.01 |
| Corticospinal tract R | 0.40 | 0.07 | 0.41 | 0.09 | 2.05 | -5.58 | 9.69 | 0.59 |
| Corticospinal tracl L | 0.36 | 0.05 | 0.37 | 0.08 | 3.11 | -2.88 | 9.09 | 0.30 |
| SCP R | 0.36 | 0.08 | 0.38 | 0.11 | 5.58 | -2.96 | 14.11 | 0.19 |
| SCP L | 0.32 | 0.07 | 0.35 | 0.08 | 5.16 | -1.85 | 12.16 | 0.14 |
| ***Regions of interest (AX 10^-3^mm ^2^/s)*** |  |  |  |  |  |  |  |  |
| Genu Corpus Callosum | 1.59 | 0.04 | 1.62 | 0.08 | 1.43 | -3.40 | 6.25 | 0.55 |
| Body Corpus Callosum | 1.62 | 0.05 | 1.68 | 0.08 | 7.39 | 2.21 | 12.57 | 0.01 |
| Splenium Corpus Callosum | 1.63 | 0.05 | 1.67 | 0.07 | 5.28 | -0.14 | 10.70 | 0.06 |
| Cingulum (paracallosal) R | 1.32 | 0.06 | 1.27 | 0.05 | -2.86 | -7.78 | 2.05 | 0.24 |
| Cingulum (paracallosal) L | 1.28 | 0.06 | 1.23 | 0.06 | -2.46 | -8.19 | 3.28 | 0.39 |
| Cingulum (parahippocampal) R | 1.11 | 0.03 | 1.20 | 0.11 | 6.69 | -0.69 | 14.07 | 0.07 |
| Cingulum (parahippocampal) L | 1.10 | 0.04 | 1.19 | 0.11 | 9.60 | 2.44 | 16.77 | 0.01 |
| Fornix | 1.49 | 0.05 | 1.56 | 0.10 | 5.61 | 0.30 | 10.93 | 0.04 |
| Uncinate Fasciculus R | 1.11 | 0.05 | 1.22 | 0.14 | 11.04 | 1.34 | 20.73 | 0.03 |
| Uncinate Fasciculus L | 1.12 | 0.04 | 1.24 | 0.19 | 11.37 | -1.38 | 24.11 | 0.08 |
| Corticospinal tract R | 1.17 | 0.06 | 1.18 | 0.11 | 1.41 | -6.79 | 9.61 | 0.73 |
| Corticospinal tracl L | 1.14 | 0.06 | 1.12 | 0.14 | 1.13 | -8.99 | 11.25 | 0.82 |
| SCP R | 1.85 | 0.10 | 1.85 | 0.11 | 5.07 | -4.25 | 14.40 | 0.28 |
| SCP L | 1.69 | 0.10 | 0.80 | 0.07 | 2.33 | -6.99 | 11.64 | 0.61 |

**Supplementary Table 2.** Baseline DTI metric data for individual white matter regions of interest for control participants and patients. AX, Axial Diffusivity; EMD, Estimated Mean Difference; RD, Radial Diffusivity; SCP, Superior Cerebellar Peduncle. *Linear regression comparing bvFTD (n=19) with controls after adjusting for age, gender and disease duration.

|  | **bvFTD (n=19)** | | | | **bvFTD - MAPT (n=8)** | | | | **bvFTD - Sporadic (n=7)** | | | | **bvFTD -C9ORF72 (n=4)** | | | |
| --- | --- | --- | --- | --- | --- | --- | --- | --- | --- | --- | --- | --- | --- | --- | --- | --- |
| ***Region of interest* (RD**) | %/year change | CI 95% | | p-value | %/year change | CI 95% | | p-value | %/year change | CI 95% | | p-value | %/year change | CI 95% | | p-value |
| Genu CC | 4.8 | -2.2 | 13.5 | 0.2 | 4.8 | -4.5 | 16.8 | 0.3 | 6.4 | -0.3 | 14.5 | 0.1 | -2.5 | -10.6 | 6.9 | 0.7 |
| Body CC | 5.8 | 1.9 | 9.0 | 0.002 | 7.4 | 3.4 | 10.6 | <0.001 | 3.4 | -1.5 | 7.8 | 0.2 | 6.2 | -0.6 | 12.3 | 0.1 |
| Splenium CC | 7.3 | 2.5 | 10.1 | 0.001 | 7.3 | 1.5 | 11.1 | 0.01 | 6.8 | 1.3 | 10.2 | 0.01 | 8.7 | -0.1 | 15.3 | 0.1 |
| Cingulum (paracallosal) R | 8.6 | 2.8 | 11.6 | 0.001 | 7.7 | 0.3 | 12.2 | 0.04 | 9.5 | 3.0 | 13.0 | 0.002 | 7.8 | -1.2 | 13.9 | 0.1 |
| Cingulum (paracallosal) L | 6.9 | 2.5 | 10.0 | 0.001 | 6.9 | 1.4 | 11.2 | 0.01 | 8.0 | 2.9 | 11.7 | 0.001 | 3.4 | -3.6 | 9.1 | 0.4 |
| Cingulum (parahippocampal) R | 2.5 | 0.9 | 8.3 | 0.01 | 3.7 | 1.8 | 9.7 | 0.01 | 0.9 | -1.9 | 7.9 | 0.2 | 3.6 | -2.5 | 14.3 | 0.2 |
| Cingulum (parahippocampal) L | 4.8 | 0.8 | 11.4 | 0.03 | 8.7 | 4.7 | 15.6 | <0.001 | -0.2 | -5.3 | 7.5 | 0.7 | 2.1 | -7.5 | 15.1 | 0.5 |
| Fornix | 1.6 | -1.8 | 7.8 | 0.2 | 1.7 | -2.9 | 9.7 | 0.3 | 2.2 | -1.4 | 8.3 | 0.2 | -2.9 | -6.9 | 3.6 | 0.5 |
| Uncinate Fasciculus R | 5.6 | 3.0 | 10.7 | <0.001 | 7.4 | 4.7 | 12.6 | <0.001 | 4.3 | 0.5 | 10.6 | 0.03 | 3.3 | -3.2 | 12.4 | 0.2 |
| Uncinate Fasciculus L | 7.4 | 2.0 | 13.8 | 0.01 | 12.7 | 6.3 | 20.6 | <0.001 | 0.5 | -2.8 | 4.6 | 0.6 | **8.2** | **1.4** | **16.1** | **0.02** |
| Corticospinal tract R | 3.0 | -5.1 | 14.3 | 0.4 | 4.5 | -6.7 | 19.0 | 0.3 | 4.9 | -5.9 | 18.6 | 0.3 | -7.0 | -24.0 | 13.1 | 0.6 |
| Corticospinal tracl L | 4.9 | -1.7 | 15.9 | 0.1 | 5.7 | -3.7 | 19.6 | 0.2 | 2.3 | -7.1 | 15.7 | 0.5 | 11.4 | -5.3 | 32.3 | 0.2 |
| SCP R | 3.0 | -5.1 | 14.3 | 0.4 | 4.5 | -6.7 | 19.0 | 0.3 | 4.9 | -5.9 | 18.6 | 0.3 | -7.0 | -24.0 | 13.1 | 0.6 |
| SCP L | 6.3 | -0.8 | 19.8 | 0.1 | 8.1 | -0.5 | 23.2 | 0.1 | 4.6 | -5.0 | 20.8 | 0.2 | 2.2 | -18.1 | 30.3 | 0.6 |
| ***Region of interest* (AX**) |  |  |  |  |  |  |  |  |  |  |  |  |  |  |  |  |
| Genu CC | 0.9 | -0.8 | 3.0 | 0.2 | 0.3 | -2.0 | 3.4 | 0.6 | 1.4 | 0.1 | 3.0 | 0.03 | -0.4 | -2.3 | 1.8 | 0.8 |
| Body CC | 1.2 | -0.2 | 1.8 | 0.1 | 1.2 | -0.3 | 1.9 | 0.1 | 0.6 | -1.2 | 1.7 | 0.8 | **2.8** | **0.8** | **3.9** | **0.003** |
| Splenium CC | 1.2 | 0.2 | 2.5 | 0.02 | 1.4 | 0.1 | 3.0 | 0.04 | 0.8 | -0.4 | 2.3 | 0.2 | 1.2 | -0.8 | 3.6 | 0.2 |
| Cingulum (paracallosal) R | 0.1 | -1.4 | 1.0 | 0.8 | 0.1 | -1.8 | 1.5 | 0.9 | 0.4 | -1.3 | 1.7 | 0.8 | -1.1 | -3.9 | 1.1 | 0.3 |
| Cingulum (paracallosal) L | -0.1 | -1.3 | 1.4 | 0.9 | -0.6 | -2.2 | 1.2 | 0.6 | 0.0 | -1.9 | 2.1 | 0.9 | 1.0 | -1.4 | 3.6 | 0.4 |
| Cingulum (parahippocampal) R | 3.3 | 2.0 | 5.9 | <0.001 | 4.7 | 3.2 | 7.4 | <0.001 | 2.8 | 0.8 | 6.0 | 0.01 | 2.8 | -1.2 | 7.7 | 0.1 |
| Cingulum (parahippocampal) L | 3.2 | 1.2 | 6.5 | 0.005 | 5.4 | 3.5 | 8.6 | <0.001 | 1.0 | -1.6 | 4.7 | 0.3 | 1.4 | -2.6 | 6.7 | 0.4 |
| Fornix | 0.4 | -0.8 | 2.4 | 0.3 | -0.1 | -1.7 | 2.7 | 0.6 | 0.8 | -0.4 | 2.7 | 0.1 | -0.8 | -2.3 | 1.5 | 0.7 |
| Uncinate Fasciculus R | 2.6 | 1.3 | 5.1 | 0.001 | 3.8 | 2.2 | 6.6 | <0.001 | 1.9 | 0.3 | 4.7 | 0.03 | 0.8 | -2.4 | 5.3 | 0.5 |
| Uncinate Fasciculus L | 4.7 | 1.1 | 7.7 | 0.009 | 8.0 | 3.7 | 12.0 | <0.001 | 0.8 | -1.2 | 2.2 | 0.6 | **4.1** | **0.4** | **7.3** | **0.03** |
| Corticospinal tract R | -0.1 | -2.7 | 3.4 | 0.8 | 0.6 | -3.0 | 5.0 | 0.6 | -0.7 | -4.4 | 3.8 | 0.9 | 1.0 | -5.1 | 8.1 | 0.7 |
| Corticospinal tracl L | 0.8 | -1.7 | 5.2 | 0.3 | 1.0 | -2.4 | 6.5 | 0.4 | -0.7 | -4.1 | 4.4 | 0.9 | 4.5 | -1.7 | 12.4 | 0.1 |
| SCP R | -0.1 | -2.7 | 3.4 | 0.8 | 0.6 | -3.0 | 5.0 | 0.6 | -0.7 | -4.4 | 3.8 | 0.9 | 1.0 | -5.1 | 8.1 | 0.7 |
| SCP L | 2.9 | 0.8 | 5.6 | 0.008 | 2.8 | 0.4 | 5.8 | 0.03 | 2.2 | -0.4 | 5.4 | 0.1 | 6.8 | 2.4 | 11.8 | 0.003 |

**Supplementary Table 3.** Estimated percentage per year difference in the rate of change of Radial Diffusivity (RD) and Axial Diffusivity (AX) for bvFTD patients and bvFTD subgroups, by region, compared with controls. bvFTD, behavioural variant Frontotemporal Dementia; CI, Confidence interval; CC, corpus callosum; SCP, superior cerebellar peduncle.
